# Deprescribing interventions and their impact on medication adherence in community-dwelling older adults with polypharmacy: a systematic review

**DOI:** 10.1186/s12877-019-1031-4

**Published:** 2019-01-18

**Authors:** Joanna Ulley, Deborah Harrop, Ali Ali, Sarah Alton, Sally Fowler Davis

**Affiliations:** 1 Sheffield Teaching Hospitals NHS Foundation Trust, Sheffield, England; 2 Sheffield Hallam University, Montgomery House, 32 Collegiate Crescent, Sheffield, S10 2BP England

**Keywords:** Polypharmacy, Deprescribing, Adherence, Systematic review, Older person

## Abstract

**Background:**

Polypharmacy, and the associated adverse drug events such as non-adherence to prescriptions, is a common problem for elderly people living with multiple comorbidities. Deprescribing, i.e. the gradual withdrawal from medications with supervision by a healthcare professional, is regarded as a means of reducing adverse effects of multiple medications including non-adherence. This systematic review examines the evidence of deprescribing as an effective strategy for improving medication adherence amongst older, community dwelling adults.

**Methods:**

A mixed methods review was undertaken. Eight bibliographic database and two clinical trials registers were searched between May and December 2017. Results were double screened in accordance with pre-defined inclusion/exclusion criteria related to polypharmacy, deprescribing and adherence in older, community dwelling populations. The Mixed Methods Appraisal Tool (MMAT) was used for quality appraisal and an a priori data collection instrument was used. For the quantitative studies, a narrative synthesis approach was taken. The qualitative data was analysed using framework analysis. Findings were integrated using a mixed methods technique. The review was performed in accordance with the PRISMA reporting statement.

**Results:**

A total of 22 original studies were included, of which 12 were RCTs. Deprescribing with adherence as an outcome measure was identified in randomised controlled trials (RCTs), observational and cohort studies from 13 countries between 1996 and 2017. There were 17 pharmacy-led interventions; others were led by General Practitioners (GP) and nurses. Four studies demonstrated an overall reduction in medications of which all studies corresponded with improved adherence. A total of thirteen studies reported improved adherence of which 5 were RCTs. Adherence was reported as a secondary outcome in all but one study.

**Conclusions:**

There is insufficient evidence to show that deprescribing improves medication adherence. Only 13 studies (of 22) reported adherence of which only 5 were randomised controlled trials. Older people are particularly susceptible to non-adherence due to multi-morbidity associated with polypharmacy. Bio-psycho-social factors including health literacy and multi-disciplinary team interventions influence adherence. The authors recommend further study into the efficacy and outcomes of medicines management interventions. A consensus on priority outcome measurements for prescribed medications is indicated.

**Trial registration:**

PROSPERO number CRD42017075315.

**Electronic supplementary material:**

The online version of this article (10.1186/s12877-019-1031-4) contains supplementary material, which is available to authorized users.

## Background

The management of many chronic diseases depends chiefly on the assumption that the medications prescribed by the clinician are taken by the individual being treated. Medication adherence is defined as the extent to which prescribed medications are taken according to the dosage and frequency recommended by the provider [1]. It is estimated, however, that between 30 and 50% of people do not take their medications as prescribed [2]. Theories as to the causes of non-adherence generally recognise socio-economic, healthcare system, condition- and patient-related factors [3]. Methods to combat non-adherence are wide-ranging; from practical interventions, to behaviour change interventions targeting psychological barriers to medication use [4]. The resulting costs of non-adherence are both human, the individual not gaining the benefit of treatment, and economic [5–7], resulting in unused medication, for example. Older people are particularly susceptible to non-adherence due to multi-morbidity associated polypharmacy [8], and owing to particular constraints such as physical, cognitive or sensory impairments [9].

In this review polypharmacy is the taking of any medication that is potentially inappropriate, rather than exceeding a defined number of drugs [10] because adherence to an inappropriate medication can lead to harm. However, available evidence regarding polypharmacy and drug adherence in the older population living at home is scarce [11]. Most studies have focused on improving adherence to one drug group, and thus have limited applicability to the older population who commonly use multiple medications [12–14].

The term ‘deprescribing’ is the process of the gradual withdrawal and cessation of potentially inappropriate medications, supervised by a healthcare professional with the goal of reducing unnecessary medications and their related problems [15]. Deprescribing attempts to balance the potential for benefit and harm by systematically withdrawing inappropriate medications with the goal of managing polypharmacy and improving outcomes [16]. Guidelines that have been developed to facilitate this process advocate involving the individual themselves in decisions relating to their prescription [17–20]. In spite of their potential to improve safety, significant barriers to these interventions exist [21–23].

Previous studies have examined the impact of deprescribing processes in residential care settings [24]. To the best of the authors’ knowledge, there has been no investigation as to the potential of deprescribing as an effective strategy for improving medication adherence in community-dwelling older adults. This is a population who are largely independent and live in their own home, but for whom the number, dosage and complexity of medications demands considerable health literacy [25, 26].

The aim of the review was to explore the impact of deprescribing interventions on adherence measures in this unique population.

## Methods

A mixed methods systematic review of the literature was undertaken on deprescribing interventions and medication adherence in community-dwelling older adults with polypharmacy. Registered as PROSPERO CRD42017075315, this systematic review used qualitative and quantitative data to explore a range of factors associated with the effectiveness of deprescribing interventions in tackling medication non-adherence, and in doing so, offers a more complete picture of the area of investigation [27]. The search, screening, quality appraisal and data extraction processes were led by an Information Scientist (DH) and the lead for the review team (JU). This systematic review is reported in accordance with the Preferred Reporting Items for Systematic review and Meta-Analysis (PRISMA) statement.

### Eligibility criteria

Papers were selected using the criteria as follows. Study type: primary qualitative, quantitative or mixed methods studies were sought. Protocols, conference abstracts, academic thesis, editorials, commentaries and opinion articles were excluded. Review papers were excluded, but were used to cross-check for relevant primary papers. Population: papers must have reported data on community dwelling older people (aged ≥65 years) experiencing polypharmacy. In this review, polypharmacy is the taking of any medication that is potentially inappropriate, rather than exceeding a defined number of drugs [10]. Intervention: a paper must have explored the effect of a deprescribing intervention which included any medication review that was in line with the definition agreed by the Pharmaceutical Care Network Europe 2013. [28]. Comparator: a study must have included a control to have been eligible for inclusion. Outcomes: a paper must have reported data on adherence, measured by any means; i.e., pill count, self-reported, questionnaire, ‘drug or prescription or medications’ ‘renewal or refill’, blood level analysis. Adherence could have been reported as a primary or a secondary outcome. Any study that did not report adherence was excluded.

### Search strategy

An information scientist (DH) undertook a comprehensive search of eight bibliographic databases between May and August 2017: ASSIA (ProQuest), CENTRAL (Wiley), CINAHL (EBSCO), MEDLINE (EBSCO), PsycINFO (ProQuest), Scopus (Elsevier), Sociological Abstracts (ProQuest), Web of Science (Thomson Reuters). Two clinical trials registers were searched in December 2017: UK Clinical Trials Gateway (NHS, National Institute for Health Research), International Clinical Trials Registry Platform (World Health Organization).

The search strategy comprised four facets: (i) older people, and (ii) polypharmacy, deprescribing or medications reviews, and (iii) adherence or instruments/measurements relating to adherence, and (iv) the community setting, such as independent living, or the community based professional delivering the intervention. All terms were searched for in the title and abstract fields and controlled vocabulary terms were included where available. The Boolean operators AND and OR were used, alongside truncation, phrase searching and proximity operators. Only papers published in the English language were sought, no date limits were applied. The search syntax was adapted for use on each information source. The full search strategy, written up for MEDLINE (EBSCO) is provided in Additional file 1: Appendix 1.

### Data management

The bibliographic software, RefWorks (ProQuest) was used to store and organise all results from the bibliographic databases, and Excel was used for results from the clinical trials registers. Microsoft Excel 2015 was used to support the selection of papers and data extraction process, and Microsoft Word 2015, the quality appraisal process.

### Study selection

All papers retrieved from the literature searches were assessed against the inclusion criteria. The study selection process was piloted by all members of the review team using a sample of 100 papers. All results were independently screened by two members of the review team. In the first instance the title and abstract of all papers were screened to determine their relevancy, followed by a full text screening of all remaining papers. Any disagreement in screening outcome between reviewers was resolved through discussion or the use of a third reviewer. Reviewers were not blinded to a paper’s author/s.

### Data extraction

An a priori data collection instrument was created and piloted by the review team. Data extraction was undertaken by one reviewer and double checked for accuracy by a second. Any discrepancies were resolved through discussion and further scrutiny of the included paper. Extracted data were as follows: information on the author/s, year of publication, name of paper, title of journal; the study setting, duration and geographic location; the study type, design, aim, objectives, context, and data collection and analysis techniques; the number of participants, participant demographics, e.g. age, gender, baseline cognitive function, number of medications taken, health conditions experienced; the aim and description of the intervention, the intervention instrument, baseline adherence, how the intervention was delivered; time from the intervention to the follow-up; theoretical framework underpinning an intervention; author stated strengths/weaknesses and conclusions; reviewers comments. Outcomes of adherence included: (i) the adherence measure reported, (ii) the number of drugs prescribed and their appropriateness, (iii) and whether the adherence measure improved with intervention or not. Secondary measures of interest included: self-reported quality of life, primary care visits, hospital visits and mortality; indicators of acceptability to users, and evidence of shared decision making.

### Risk of bias in individual studies

The Mixed Methods Quality Appraisal Tool (MMAT) [29] was used to explore the risk of bias at study level. An overall quality score was attributed to each study. An incremental score of 25% is given for each of the four criteria met for quantitative and qualitative studies and each of the three criteria for mixed methods studies.

### Data synthesis

Qualitative and quantitative data were analysed separately. If a paper reported mixed methods data, a decision was taken as to what the majority of the data comprised. The quantitative data was not homogenous; there was variability in data collection, data source, and statistical analysis across most studies. Therefore for the quantitative studies, a narrative synthesis approach was taken. The qualitative data was analysed using Framework Analysis [30]. Findings were integrated using the ‘Following a Thread’ technique described by O’Cathain et al., [31]. Using this approach all data sets were scanned for key themes and questions in need of further exploration.

## Results

**Fig. 1:**
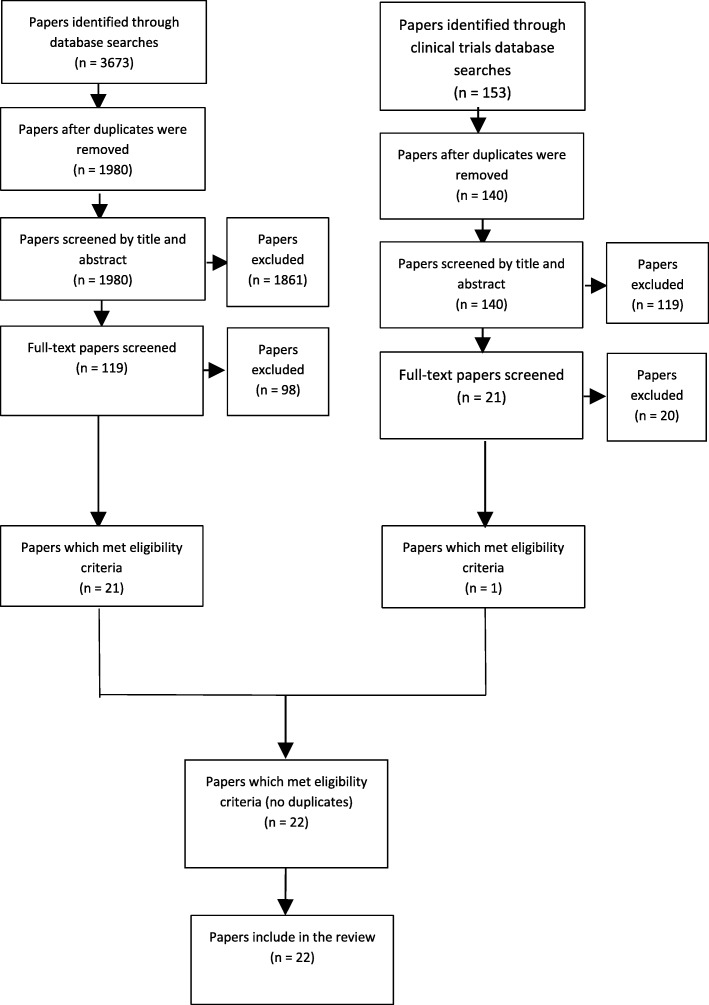
PRISMA flow diagram

A total of 1980 unique papers were yielded from the database searches, and an additional 140 papers from the clinical trials registers searches. Screening title and abstracts resulted in 119 papers being retained from the database searches and 21 papers from the clinical trials registries. After full-text reading, author citation and reference list evaluation a total of 22 met the eligibility criteria and underwent quality appraisal and data extraction processes. Nine additional trials were found, but no data was found due to either non-publication or ongoing research. The literature review screening process is summarised in Fig. 1.

Outcomes from the MMAT exercise for the 22 papers from the database searches showed that ten studies scored 100%, six scored 75% and six scored 50% or less. Non-RCT data exhibited somewhat greater risk of bias (see MMAT summary Table 1). Quantitative and qualitative findings were collated but the framework analysis was limited by the very small amount of qualitative data about adherence, medicines’ management methods and patient outcomes. A summary of study characteristics are presented in Table 2.

**Table 1:** Summary of MMAT

	Criteria	2.1 Randomisation	2.2 Concealment /blinding	2.3 Complete outcomes	2.4 Loss to follow up	3.1 Recruitment	3.2 appropriate measures	3.3 Comparable groups	3.4 Complete outcomes	4.1 Relevant sampling	4.2 Representative sampling	4.3 Appropriate measures	4.4 Response rate	Overall score
Study														
RCTs														
Basheti 2016 [34]		√	√	√	√									100%
Beer 2011 [52]		√	X	√	X									50%
Campins 2017 [32]		√	√	√	√									100%
Grymonpre 2001 [45]		√	√	X	√									75%
Haag 2016 [33]		√	√	√	√									100%
Lowe 2000 [47]		√	√	√	√									100%
Hanlon 1996 [48]		√	√	√	√									100%
Hedegaard 2015 [36]		√	√	√	X									75%
Jager 2017 [50]		√	√	√	X									75%
Sturgess 2003 [44]		√	X	X	X									25%
Vinks 2009 [43]		√	√	√	√									100%
Messerli 2016 [53]		√	√	√	√									100%
Average														83.3%
Non-RCTs														
Chen 2016 [35]						√	√	X	√					75%
Fiss 2013						√	X	X	√					50%
Griffiths 2004 [51]						√	√	X	X					50%
Lee 2015 [38]						√	√	√	√					100%
Steele 2016 [37]						X	√	√	√					75%
Hatah 2014 [41]						X	X	X	√					25%
Tan 2014 [40]						X	√	X	X					25%
Twigg 2015 [39]										√	√	√	√	100%
Raynor 2000 [46]										√	√	X	√	75%
Roth 2013 [42]										√	√	√	√	100%
Average														67.5%

**Table 2:** Summary of study characteristics

Ref	Study design	Setting	Study duration (months)	Total number of participants (*N* = intervention group where relevant)	Type of intervention/ person leading	Usual care (if applicable)	Adherence outcome
Basheti 2016 Jordan	RCT	Outpatient Clinic	3	112 (*N* = 50)	Follow up Pharmacist led medication review	Routine clinical practice	Reduction in self- reported non-adherence in intervention group (*P* < 0.001) compared with control group (*P* = 0.168)
Beer 2011 Australia	RCT	Home setting and residential aged-care facility	Unknown	30 (*N* = 15)	Physician led targeted medication withdrawal	Usual care	No significant difference between the groups *P* = 0.17
Campins 2017 Spain	RCT	Primary care centres	12	503 (*N* = 252)	Pharmacist led medication review	Routine clinical practice	At 6 months adherence was higher in the intervention group (76.4% v 64.1%) *P* = 0.005
Grymonpre 2001 Canada	RCT	Community based clinic	Variable (from baseline to follow up letter)	135 (*N* = 69)	Pharmacist led medication review	Routine clinical practice	No significant impact on adherence from baseline to follow-up (*P* = 0.895)
Haag 2016 USA	RCT	Primary care outpatient clinic	1	25 (*N* = 13)	Pharmacist led medication review	Pre-existing out-patient care transition programme	No significant difference in adherence *P* = 0.65
Hanlon 1996 USA	RCT	General Medicine Clinic at Veterans Affairs Medical Centre	12	208 (*N* = 105)	Pharmacist led medication review	Usual care	No significant difference in medication compliance (*P* = 0.88)
Hedegaard 2015 Denmark	RCT	Outpatient clinics	12	532 (*N* = 240)	Pharmacist led medication review	Routine clinical practice	Trend toward improved adherence at 3, 9 and 12 months. Greater % of control group non-adherent compared with intervention group 30.2% vs 20.3% *P* = 0.01.
Jaeger 2017 Germany	RCT	GP Practices	9	273 (*N* = 143)	Tailored medication review programme delivered by GPs and Health Care Assistants	Routine clinical practice	No significant effects on adherence *P* = 0.11
Lowe 2000 UK	RCT	General Practice / Home setting	3	161 (*N* = 77)	Pharmacist led review	Routine clinical practice	Significant difference in mean compliance score (tablet count and self –reported). Intervention group = 91.3% vs 79.5% control group. *P* < 0.001
Sturgess 2003 Northern Ireland	RCT	Community Pharmacies	18	191 (*N* = 110)	Community Pharmacist intervention programme	Routine Practice	Significant increase in compliance and fewer problems with medication compared with control group (*P* < 0.05)
Vinks 2009 Netherlands	RCT	Community Pharmacy	4	174 (*N* = 87)	Community Pharmacist review	Usual Practice	Significant reduction in the number of drug related problems per patient (includes non-compliance) -16.3% (−24.3,-8.3) 95% CI
Messerli 2016 Switzerland	RCT	Community Pharmacy	7	450 (*N* = 218)	Community Pharmacist Polymedication Check (PMC)	Routine practice	No significant difference in adherence between the two groups could be observed (*p* = 0.817)
Chen 2016 Taiwan	Prospective cross-sectional	Outpatient clinics	3	152	Pharmacist led medication therapy management service		Increase in medication adherence (MMAS-4 scale) from 3.02 to 3.92 (*p* < 0.001)
Fiss 2013 Germany	Prospective cohort	Ambulatory primary healthcare	1–24 (mean = 9)	911 (*N* = 393)	Pharmaceutical care from local pharmacy plus medical intervention by GP		Increased in adherence forgetfulness *P* = 0.001 Increased adherence deliberate *p* = 0.003 (*n* = 400) between baseline and follow up
Griffiths 2004 Australia	Cohort	Community / Home setting	1	*N* = 24	Community nurse medication review	Routine clinical practice	No significant difference in non-adherence pre and post intervention (*P* = 0.237)
Hatah 2014 New Zealand	Retrospective cohort	Community Pharmacy	6 to 41	*N* = 353	Community Pharmacist Medicines Use Review (MUR)		No significant difference except during the third visit where more patients with lower adherence scores did not return *P* < 0.001
Lee 2015 Hong Kong	Prospective Uncontrolled	Community outreach	8	*N* = 103	Pharmacist led review	Routine clinical practice	Significant reduction in Morisky Medication Adherence score *P* = 0.005
Raynor 2000 UK	Cohort	Community Pharmacy/home setting	2	*N* = 143	Community Pharmacist led medication adherence support	Routine practice	Non-adherence fell from 38% to 14% (*P* < 0.001)
Roth 2013 USA	Prospective	Community based primary care medical practice	6	64	Clinical Pharmacist led medication review	Routine clinical practice	Significant reduction in the number of medication related problems per patient (*P* < 0.001) which included non- adherence
Steele 2016 USA	Prospective Study	Home based	3	25	Pharmacist conducted home based medication review	Routine practice	Non- adherence was significantly reduced (*P* = 0.012)
Tan 2014 Australia	Prospective Study	Community clinic/ home setting	6	82	Pharmacist led review	Routine practice	Significant improvement in adherence (44.1% v 62.7% *P* = 0.023)
Twigg 2015 UK	Service Evaluation	Community Pharmacy	6	620	Community Pharmacist Review	Routine Practice	Significant increase in adherence 0.513 .337 to 0.689) 95% CI

### Studies and participants

Twenty-two studies in 13 different countries were included (10 in Europe, 5 in North America, 7 elsewhere (Australia, New Zealand, Taiwan, Hong Kong and Jordan), with publication dates between 1996 and 2017, involving 5118 participants. Thirteen studies were controlled clinical trials (1 non-randomised), and nine were cross-sectional studies. The age range of the participants was 46 and 97 years. Three studies included patients < 65 years, 13 studies age range 65–85, 6 studies included patients >86y. Eight study interventions were based in primary care or GP practices, seven in community pharmacies, three in an outpatient clinic, two as home visits, one as remote prescribing and one from a community centre for older people. The follow-up period for the interventions ranged between four weeks and 41 months, with a mean of eight months.

### Types of interventions

The healthcare professional leading the intervention varied significantly. Most commonly, a pharmacist led (in 18 studies [32–48], while a GP and pharmacist co-led in 1 study [49], a GP alone in another study [50], a community nurse led in another study [51] and the participants themselves led in another [52]. While all interventions included a process of deprescribing, study interventions used ‘deprescribing’ specifically in nine studies [32, 35, 40, 42, 47, 48, 52, 53], while in 12 studies the description was ‘medication use review’ [33, 34, 36–38, 41, 43–46, 49–51].

Where specific deprescribing tools were used they included the GP-GP (good palliative-geriatric practice) [32], STOPP/START, 2 [32, 39], Beers, 1 [52], e-medicines information, one [35], IMAP (individualised medication assessment and planning program), 1 [42]. In four studies no particular deprescribing tool was mentioned [40, 47, 48, 52]. Only four of the 22 studies yielded significant reductions in tablet burden [32, 46, 47, 50]. Regime complexity, i.e. the frequency and dosing, was not recorded in any of the studies. Of those studies with no decrease in overall medications; seven demonstrated a comparable number pre- and post-intervention [33, 36, 42, 43, 45, 48, 52]; 1 study reported an overall increase [44], and nine did not record the numbers (either pre or post) [34, 35, 37–41, 49, 51, 53].

### Medication adherence

Thirteen studies reported improved adherence following study interventions [32, 34–41, 43, 46, 47, 50] of which 5 studies were RCTs. Of the four studies that report a reduction in medication burden, all reported improved adherence [32, 46, 47, 50]. Exclusion of studies at moderate to high risk of bias (MMAT score 50% or less) did not alter the overall findings. The adherence tool varied between studies, but not to the same extent as the intervention tool. The Morisky-Green scale or a derivative was used in 10 studies [32–35, 38–40, 42, 46, 49, 52]; Medication possession ratio/pill count in five studies [36, 37, 43, 45, 49, 52]; self-report only in three studies [48, 50, 51] and self-report and pill count in three studies [44, 47, 50]. An unspecified adherence scale was used in one study [41]. All studies reported adherence as a secondary outcome, except Twigg [39] and Messerli [53]. The proposed mechanisms of success for those that reported improved adherence were: reduced number of medications, 1 [32]; good relationship between clinical professionals/ collaborative working, 3 [34, 43, 50]; practical aspect only, 1 [45]; educational or motivational impact, 5 [36, 38–41], both practical and behavioural impact, 3 [35, 37, 47].

In terms of the secondary measures of interest, self-reported quality of life was reported in five of the 22 studies [32, 34, 39, 44, 48], of which one study [39] reported an improvement. The other four studies had no statistical impact. Primary care visits were reported in four studies [32, 39, 40, 44] of which one study reported a change in number of visits [39] (an increase from a mean of 1.65 to 2.04 visits per patient in a 6 month period). Unplanned or emergency care visits were reported in 6 studies [32, 33, 39, 40, 42, 44]. Two studies suggested that interventions resulted in reductions to unplanned care but this was not statistically significant; four studies demonstrated no impact on unplanned or emergency care. Mortality was reported in four studies [32, 33, 44, 45]; none of these reported any impact.

## Discussion

The issue of adherence is manifestly complicated. A broad range of studies was deliberately included to enhance understanding of the potential mechanisms of any intervention effect but qualitative data were limited. It is clear that no routine or consistent method of deprescribing is implemented, and no consistent outcome from the practice, such as a reduction in the number of prescriptions, is reported. Therefore it was impossible to demonstrate that an improved adherence was a consequence of a reduced medication burden.

Adherence is regarded as an important indicator of the quality of the doctor–patient relationship [54] and by extension that offered by other multidisciplinary team members. It is important to note that there are different understandings of adherence according to clinical and research priorities. This study identified that improved adherence depended on the measurement tool and the method of statistical analysis. For example, a self-report scale questionnaire would differ in analysis to the proportion of days covered. One study incorporated the understanding of the individual as to the purpose of each of their medications [41], where others merely counted the number of pills left at the end of a month [36, 37, 43, 47].

There is an extremely mixed body of literature reporting strategies to improve medication adherence [55]. Adherence was measured as a secondary outcome in all but two studies which meant that more subtle reasons for non-adherence could not be established from this review. Follow-up periods were generally short, making it difficult to draw any conclusions as to the lasting impact of the interventions themselves.

The range of professional practitioners engaged in deprescribing implies that international models of practice are continuing to adopt an interdisciplinary team approach. Of the three studies that did not involve pharmacists, none showed a difference in adherence measures. Of the 18 remaining studies, the majority (13 studies) demonstrated improved outcomes. From this review it would seem that pharmacist-led deprescribing interventions are effective.

Independent prescribing status in the UK has been awarded by the General Pharmaceutical Council since 2006 and nurses, podiatrists, physiotherapists and therapeutic radiographers may complete additional post-registration training to become independent prescribers [56]. One study commented that the success of the intervention depended, in large part, on the physician’s acceptance of the recommended changes in a person’s medications [34]. Collaborative working between professionals to achieve deprescribing or increased adherence may be key to the success of these interventions.

A systematic method of enhancing the quality of research into medication use is now being proposed, particularly focusing on seven factors that are consistent with the findings of this study; vulnerable patient populations; polypharmacy and multi-morbidity; person-centered practice and research; deprescribing or medicines use review; methodological development; variability in medication use; and national and international comparative research [56].

From this review there is little data suggesting that deprescribing interventions improve outcomes such as quality of life, primary or secondary care visits or affect mortality [57]. On the other hand, neither is there much evidence that these interventions lead to a negative impact on these measures; something that has already been suggested elsewhere [58]. Large RCTs evaluating multidisciplinary interventions and clinical outcomes of deprescribing are lacking [59–61], but there are examples of individual studies suggesting that deprescribing can improve outcomes such as falls, hospital admissions and mortality [62].

There was a surprisingly low level of reporting of socio-economic components. In a sub group analysis of four studies that reported both deprescribing with reduction in medication and an improvement in adherence the intervention cohort (*N* = 847) were 44% men and 56% women. The studies reported inconsistently, on marital status educational level and household income. Further consideration of these socio-economic components is indicated to deliver a patient-centred deprescribing process [63, 64].

The strengths of this review include careful attention to screening and data extraction of the adherence outcome and the focus on the outcome of the complex medicines management intervention. ‘Deprescribing’ was carefully defined to include all forms of medication review interventions where the intention was to reduce treatment burden.

The evidence found was from different study designs: RCTs, prospective/ retrospective cohort, prospective cross-sectional, cohort and prospective uncontrolled and is a limitation of the literature. Meta-analyses of data were not possible, and this is a limitation, owing to the diversity of measures used in calculating adherence. A number of potentially interesting sub-group analyses (e.g. ethnicity, degree of frailty, economic impact of intervention) could not be completed due to varied and incomplete reporting of populations studied, interventions delivered, and outcome measurements. Qualitative data analysis revealed a number of factors associated with non-adherence but limited narrative explanation was possible within the data.

## Conclusion

This systematic review found that deprescribing as an intervention did not routinely improve medication adherence in this patient population. The theory that a reduced medication burden would improve adherence could not be substantiated in the literature. This is because the interventions described in the studies did not convincingly reduce medication burden. There is a range of bio-psycho-social factors reported that associate with improved adherence, but medicines review processes vary and rarely report the population demographic. Adherence is mostly reported as a secondary outcome and there is no standard report of successful adherence to medication. The authors recommend further study into the efficacy and outcomes of medicines management interventions. A consensus on priority outcome measurements for prescribed medications is indicated.

## Additional file

Additional file 1: Appendix 1. Search terms for the study. (DOCX 17 kb)

## Abbreviations

ASSIA: Applied Social Sciences Index and abstracts
CENTRAL: Cochrane Central Register of Controlled Trails
CINAHL: Cumulative Index to Nursing and Allied Health Literature
MEDLINE: Medical Literature
MMAT: Mixed Methods Quality Appraisal Tool
PRISMA: Preferred Reporting Items for Systematic review and Meta-Analysis
PROSPERO: Prospective Register of Systematic Reviews
PsycINFO: Psychology Informatics
RCT: Randomised Controlled Trial
USA: United States of America
