# Supplementary material for: Deprescribing interventions and their impact on medication adherence in community-dwelling older adults with polypharmacy: a systematic review
Source: BMC Geriatr. 2019 Jan 18;19:15. doi: 10.1186/s12877-019-1031-4 (PMC6339421; doi:10.1186/s12877-019-1031-4)
Supplement: Supplementary file 1 — Appendix 1. Search terms for the study. (DOCX 17 kb) [file 12877_2019_1031_MOESM1_ESM.docx]

**Appendix 1**

The search terms have been written up for MEDLINE using the EBSCO interface.

Explanation of search terms used: / = MeSH Heading; * = denotes any character/s; ti = title word; ab = abstract word; N6 = adjacency within 6 words; "" = phrase search

1. older.ti,ab
2. "over 65*".ti,ab
3. elder*.ti,ab
4. ageing.ti,ab
5. aging.ti,ab
6. senior*.ti,ab
7. geriatric*.ti,ab
8. "end of life".ti,ab
9. palliat*.ti,ab
10. terminal.ti,ab
11. pensioner*.ti,ab
12. octogenarian*.ti,ab
13. nonagenarian*.ti,ab
14. frail*.ti,ab
15. pre-frail*.ti,ab
16. "pre frail*".ti,ab
17. frail elderly/
18. aged/
19. aged, 80 and over/
20. palliative care/
21. terminal care/
22. or/1-21
23. review*.ti,ab
24. optimis*.ti,ab
25. reduc*.ti,ab
26. withdraw*.ti,ab
27. removal.ti,ab
28. remove*.ti,ab
29. taper*.ti,ab
30. discontin*.ti,ab
31. stop*.ti,ab
32. optimiz*.ti,ab
33. substitut*.ti,ab
34. or/23-33
35. deprescrib*.ti,ab
36. de-prescrib*.ti,ab
37. deprescript*.ti,ab
38. de-prescript*.ti,ab
39. polypharmacy.ti,ab
40. desprescriptions/
41. polypharmacy/
42. or/35-41
43. medication*.ti,ab
44. medicine*.ti,ab
45. prescription*.ti,ab
46. prescribe*.ti,ab
47. drug*.ti,ab
48. tablet*.ti,ab
49. pills.ti,ab
50. dose.ti,ab
51. dosage.ti,ab
52. or/43-51
53. adher*.ti,ab
54. non-adher*
55. complian*.ti,ab
56. non-complian*
57. comply*.ti,ab
58. non-comply*.ti,ab
59. concord*.ti,ab
60. non-concord*.ti,ab
61. conform.ti,ab
62. non-conform.ti,ab
63. abidance*.ti,ab
64. non-abidance*.ti,ab
65. abide*.ti,ab
66. non-abide.ti,ab
67. agreement.ti,ab
68. disagree*.ti,ab
69. accord*.ti,ab
70. non-accord.ti,ab
71. concurr*.ti,ab
72. cooperat*.ti,ab
73. uncooperat*.ti,ab
74. co-operat.ti,ab
75. unco-operat*.ti,ab
76. "pill count*".ti,ab
77. "self report*".ti,ab
78. self-report*.ti,ab
79. girerd.ti,ab
80. morisky.ti,ab
81. management.ti,ab
82. "as directed".ti,ab
83. "as instructed".ti,ab
84. "prescribed dose".ti,ab
85. "prescribed dosage".ti,ab
86. medication adherence/
87. or/53-85
88. "congregate housing".ti,ab
89. "congregate living".ti,ab
90. "supported accommodation".ti,ab
91. "supported housing".ti,ab
92. "senior housing".ti,ab
93. "extra care".ti,ab
94. "care plus".ti,ab
95. "independent housing".ti,ab
96. housebound.ti,ab
97. homebound.ti,ab
98. sheltered.ti,ab
99. "assisted living".ti,ab
100. "supported living".ti,ab
101. "retirement village*".ti,ab
102. "retirement communit*".ti,ab
103. "independent living unit*".ti,ab
104. resid* N6 home*.ti,ab
105. living N6 home*.ti,ab
106. live N6 home*.ti,ab
107. live N6 independent*.ti,ab
108. living N6 independently*.ti,ab
109. resid* N6 independent*.ti,ab
110. living N6 community.ti,ab
111. live* N6 community.ti,ab
112. dwell* N6 community.ti,ab
113. resid* N6 community.ti,ab
114. community-dwelling.ti,ab
115. home-dwelling.ti,ab
116. home* N6 dwell*.ti,ab
117. independent living/
118. housing for the elderly/
119. assisted living facilities/
120. clinician*.ti,ab
121. pharmacist*.ti,ab
122. "primary care".ti,ab
123. "primary healthcare".ti,ab
124. "general practic*".ti,ab
125. GP*.ti,ab
126. "family physician*".ti,ab
127. geriatrician*.ti,ab
128. "community-based".ti,ab
129. out-patient*.ti,ab
130. "out patient*".ti,ab
131. "community based".ti,ab
132. community-setting*.ti,ab
133. "community setting*".ti,ab
134. "community health".ti,ab
135. "community care".ti,ab
136. "family practice".ti,ab
137. "general medical practitioner*".ti,ab
138. "family doctor*".ti,ab
139. "family medical practice*".ti,ab
140. physicians, family/
141. primary health care/
142. or/88-141
143. 34 N6 52
144. 143 or 42
145. 52 N6 87
146. 145 or 86
147. 22 and 142 and 144 and 146
148. 22 and 142 and 144 and 146 English Language
